# Environmental factors regulate Paneth cell phenotype and host susceptibility to intestinal inflammation in Irgm1-deficient mice

**DOI:** 10.1242/dmm.031070

**Published:** 2018-02-01

**Authors:** Allison R. Rogala, Alexi A. Schoenborn, Brian E. Fee, Viviana A. Cantillana, Maria J. Joyce, Raad Z. Gharaibeh, Sayanty Roy, Anthony A. Fodor, R. Balfour Sartor, Gregory A. Taylor, Ajay S. Gulati

**Affiliations:** 1 Center for Gastrointestinal Biology and Disease, University of North Carolina at Chapel Hill, Chapel Hill, NC 27599, USA; 2 Department of Pediatrics, Division of Gastroenterology, University of North Carolina at Chapel Hill, Chapel Hill, NC 27599, USA; 3 Geriatric Research, Education, and Clinical Center, VA Medical Center, Durham, NC 27705, USA; 4 Departments of Medicine; Molecular Genetics and Microbiology; and Immunology; Division of Geriatrics, and Center for the Study of Aging and Human Development, Duke University Medical Center, Durham, NC 27710, USA; 5 Department of Medicine, Division of Infectious Disease, Duke University Medical Center, Durham, NC 27710, USA; 6 Department of Bioinformatics and Genomics, University of North Carolina at Charlotte, Charlotte, NC 28223, USA; 7 Department of Medicine, Division of Gastroenterology and Hepatology, University of North Carolina at Chapel Hill, Chapel Hill, NC 27599, USA; 8 Department of Microbiology and Immunology, University of North Carolina at Chapel Hill, Chapel Hill, NC 27599, USA; 9 Department of Pathology and Laboratory Medicine, University of North Carolina at Chapel Hill, Chapel Hill, NC 27599, USA

**Keywords:** Immunity-related GTPases, Experimental colitis, Inflammatory bowel diseases

## Abstract

Crohn's disease (CD) represents a chronic inflammatory disorder of the intestinal tract. Several susceptibility genes have been linked to CD, though their precise role in the pathogenesis of this disorder remains unclear. Immunity-related GTPase M (*IRGM*) is an established risk allele in CD. We have shown previously that conventionally raised (CV) mice lacking the *IRGM* ortholog, *Irgm1* exhibit abnormal Paneth cells (PCs) and increased susceptibility to intestinal injury. In the present study, we sought to utilize this model system to determine if environmental conditions impact these phenotypes, as is thought to be the case in human CD. To accomplish this, wild-type and *Irgm1^−/−^* mice were rederived into specific pathogen-free (SPF) and germ-free (GF) conditions. We next assessed how these differential housing environments influenced intestinal injury patterns, and epithelial cell morphology and function in wild-type and *Irgm1^−/−^* mice. Remarkably, in contrast to CV mice, SPF *Irgm1^−/−^* mice showed only a slight increase in susceptibility to dextran sodium sulfate-induced inflammation. SPF *Irgm1^−/−^* mice also displayed minimal abnormalities in PC number and morphology, and in antimicrobial peptide expression. Goblet cell numbers and epithelial proliferation were also unaffected by Irgm1 in SPF conditions. No microbial differences were observed between wild-type and *Irgm1^−/−^* mice, but gut bacterial communities differed profoundly between CV and SPF mice. Specifically, *Helicobacter* sequences were significantly increased in CV mice; however, inoculating SPF *Irgm1^−/−^* mice with *Helicobacter hepaticus* was not sufficient to transmit a pro-inflammatory phenotype. In summary, our findings suggest the impact of Irgm1-deficiency on susceptibility to intestinal inflammation and epithelial function is critically dependent on environmental influences. This work establishes the importance of *Irgm1^−/−^* mice as a model to elucidate host-environment interactions that regulate mucosal homeostasis and intestinal inflammatory responses. Defining such interactions will be essential for developing novel preventative and therapeutic strategies for human CD.

## Abstract (precis)

**Summary:** In this study, we rederived conventionally raised Irgm1-deficient mice into specific pathogen-free and germ-free conditions. We show that these environments determine how Irgm1 regulates Paneth cell function and gut inflammation susceptibility.

## INTRODUCTION

Crohn's disease (CD) is a chronic inflammatory disorder of the gastrointestinal tract, which is thought to occur as a result of dysregulated host immune responses to the enteric microbiota within a genetically susceptible host (Abraham and Cho, 2009; Leone et al., 2013). Genome wide association studies have identified a multitude of genes associated with development of CD, many of which encode factors involved in the regulation of the autophagy pathway (Baskaran et al., 2014; Cleynen et al., 2013; Hoefkens et al., 2013; Li et al., 2014; Liu et al., 2015; Khor et al., 2011). Among these is the immunity-related GTPase M (IRGM), which has recently been shown to play an integral role in the initiation of autophagy during microbial clearance (Chauhan et al., 2015).

We previously reported that mice deficient in Irgm1, a murine homologue of IRGM, exhibited several phenotypic differences within the gastrointestinal tract, as compared to wild-type (WT) mice (Liu et al., 2013). First, *Irgm1^−/−^* mice showed an overall increased susceptibility to dextran sodium sulfate (DSS)-induced colonic injury. Clinically, these knockout (KO) mice demonstrated greater weight loss, increased fecal blood and worsened stool consistency than WT mice treated with DSS. *Irgm1^−/−^* mice also exhibited increased inflammation in the ileum in response to DSS, which is not typically observed in this injury model (Chassaing et al., 2014). Second, we reported that *Irgm1^−/−^* mice expressed an aberrant Paneth cell (PC) phenotype. PCs are specialized epithelial cells located in the small intestine, which play an important role in innate immunity and regulation of the intestinal microbiota via secretion of antimicrobial peptides (AMPs). *Irgm1^−/−^* mice possessed an increased number of PCs per crypt, decreased PC granule size and an increased number of ectopically placed PCs. Functionally, the PCs of Irgm1-deficient mice expressed decreased mRNA levels of the AMPs α-defensin 20 (*Defa20*) and lysozyme (*Lyz*) (Liu et al., 2013).

It is important to note that mice used in the study by Liu and colleagues were born and raised in conventional (CV) conditions that did not exclude, for instance, murine norovirus or species of *Helicobacter*. Because previous work demonstrated that environmental factors, including microbial influences, can affect the penetrance and severity of intestinal inflammation in murine models of inflammatory bowel disease (IBD) (Foltz et al., 1998; Maggio-Price et al., 2006; Ward et al., 1996; Peloquin and Nguyen, 2013; Cadwell et al., 2010), we sought to determine whether environmental conditions impact the inflammatory and PC phenotypes in Irgm1-deficient mice. Specifically, we hypothesized that *Irgm1^−/−^* mice that had been rederived into ‘clean’ housing conditions have an attenuated phenotype in regards to DSS susceptibility and PC dysmorphology.

To test our hypothesis, we established colonies of littermate *Irgm1^−/−^* and WT mice in specific pathogen-free (SPF) and germ-free (GF) facilities. In this study, we demonstrate that environmental conditions remarkably attenuate the increased susceptibility of *Irgm1^−/−^* mice to DSS-mediated intestinal injury, as well as the dysmorphic PC phenotype observed in these animals. This interplay between host genetics and the environment parallels similar interactions in human CD, thereby enhancing the value of this murine model in the investigation of gene-environment interactions within the context of intestinal inflammatory disorders.

## RESULTS

### Increased susceptibility to DSS-induced intestinal injury is attenuated in *Irgm1*-deficient mice housed in SPF conditions

**Fig. 1.**
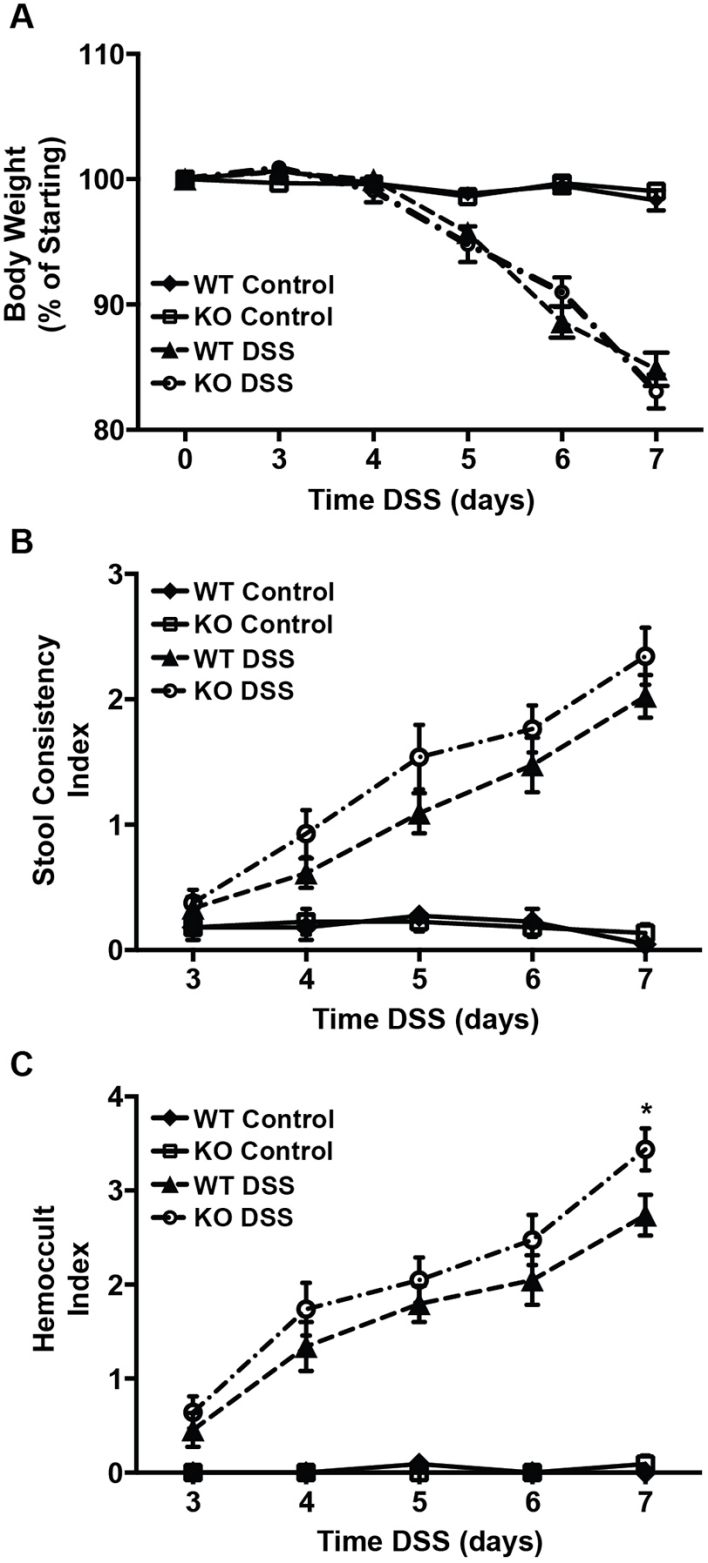
**Clinical response of *Irgm1*-deficient mice receiving dextran sodium sulfate.** Male SPF wild-type (WT) and *Irgm1*^−/−^ (KO) mice were administered 3% dextran sodium sulfate (DSS) in drinking water or drinking water alone (control) for 7 days. (A) Weight loss shown as a percentage of initial weight prior to treatment. (B) Average stool consistency score. (C) Hemoccult score measuring occult fecal blood. Values are means±s.e.m.; data are combined from four separate experiments. Combined cohort sizes are as follows: *n*=22 WT DSS, 21 KO DSS, 11 WT control, 10 KO control. *P<0.05 (KO DSS versus WT DSS).

To determine whether *Irgm1^−/−^* mice housed in SPF conditions exhibit the increased susceptibility to intestinal inflammation previously observed in CV KO animals, SPF KO and WT littermate mice were subject to acute DSS injury as described (Liu et al., 2013). In contrast to our previous findings when applying CV housing conditions, in which DSS-treated *Irgm1* KO mice lost more weight than their WT counterparts, no differences in weight loss were detected between WT and KO groups when mice were housed in an SPF environment (Fig. 1A). Similarly, no differences between *Irgm1* KO and WT mice were observed when fecal samples were scored for consistency (Fig. 1B). At 7 days post-DSS, the Hemoccult index was slightly higher in KO mice compared with their WT counterparts, but no differences in stool blood were observed at earlier time points (Fig. 1C).

**Fig. 2.**
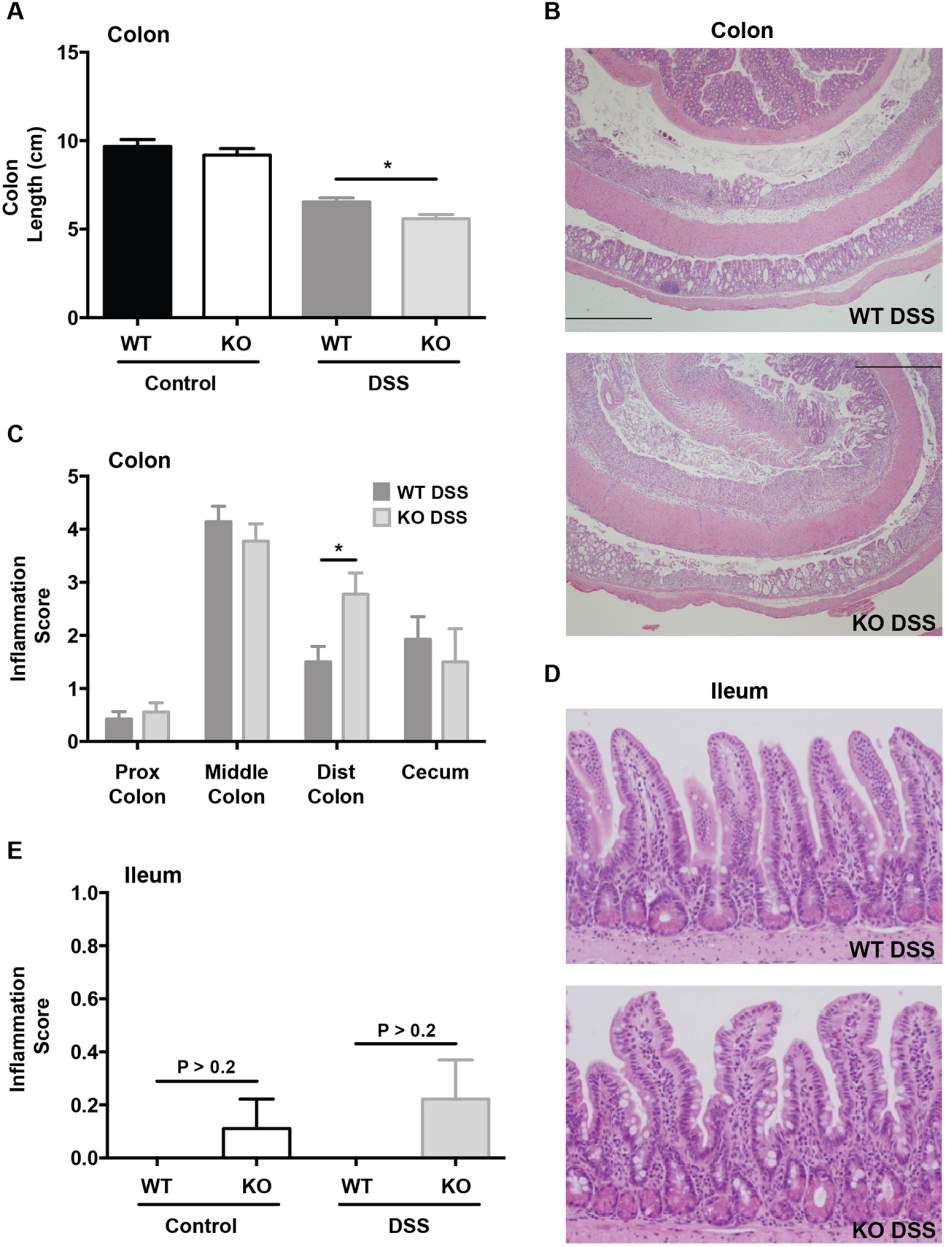
**Intestinal inflammation in specific pathogen-free *Irgm1*-deficient mice receiving dextran sodium sulfate.** Male specific pathogen-free (SPF) wild-type (WT) and *Irgm1*^−/−^ (KO) mice were administered 3% dextran sodium sulfate (DSS) in drinking water or drinking water alone (control) for 7 days. (A) Gross colon length. (B) Representative histological tissue sections from the colon of WT and KO mice treated with DSS (H&E stain, 4× magnification). (C) Average histologic inflammation scores from indicated segments of colon. (D) Representative histological tissue sections from the ileum of WT and KO mice treated with DSS (H&E stain, 20× magnification). (E) Average histologic inflammation scores in ileum. Results from water controls were omitted from B-D, as there was no inflammation present in these samples. Values are means±s.e.m.; data are combined from two separate experiments. Combined cohort sizes were as follows: *n*=14 WT DSS, 9 KO DSS, 8 WT control, 9 KO control. *P<0.01.

To assess colonic inflammation, we first measured colon lengths at the time of necropsy (after 7 days of DSS treatment). There were no baseline differences between SPF WT and *Irgm1* KO mice that received water control; however, both groups demonstrated shortened colons relative to controls when treated with DSS (Fig. 2A). This colon shortening was more pronounced in KO mice receiving DSS (*P*<0.01), though the absolute difference between DSS-treated WT and KO mice was relatively small. Histologically, the vehicle-treated controls of both the KO and WT mice lacked any discernible histologic inflammation or ulceration of the colon. Within the DSS-treated groups (Fig. 2B,C), mild inflammation occurred in the proximal colon of both KO and WT mice; however, there were no differences in severity between these two groups. Similarly, the ceca of both KO and WT DSS-treated groups showed moderate inflammation, but the genotype had no discernible effect. In the middle colon, both KO and WT groups treated with DSS exhibited an equally severe inflammatory response. The only differences in histologic inflammatory scores that was noticed in SPF mice were in the distal colon, where KO animals did display increased inflammation relative to their WT counterparts. Notably, this starkly contrasts with our previous finding in CV-raised mice, in which the genotype contributed to differences in inflammation throughout the middle colon, distal colon and cecum (Liu et al., 2013).

In the ileum, we found no significant differences in inflammation between DSS-treated WT and KO mice raised in SPF conditions (Fig. 2D,E). Ileal tissues were also assessed for expression of TNFα mRNA; however, neither *Irgm1* KO nor WT mice housed in SPF conditions yielded detectable levels of TNFα mRNA as compared to positive controls (data not shown). Again, this contrasts our previous findings in CV KO mice, which showed increased ileal inflammation and TNFα mRNA expression in response to DSS, as compared to their WT littermates (Liu et al., 2013). In summary, the increased susceptibility to ileal and colonic inflammation in response to DSS that is displayed by CV *Irgm1*-deficient mice is markedly attenuated in SPF housing conditions.

### Environmental conditions influence the PC phenotype in *Irgm1*-deficient mice

**Fig. 3.**
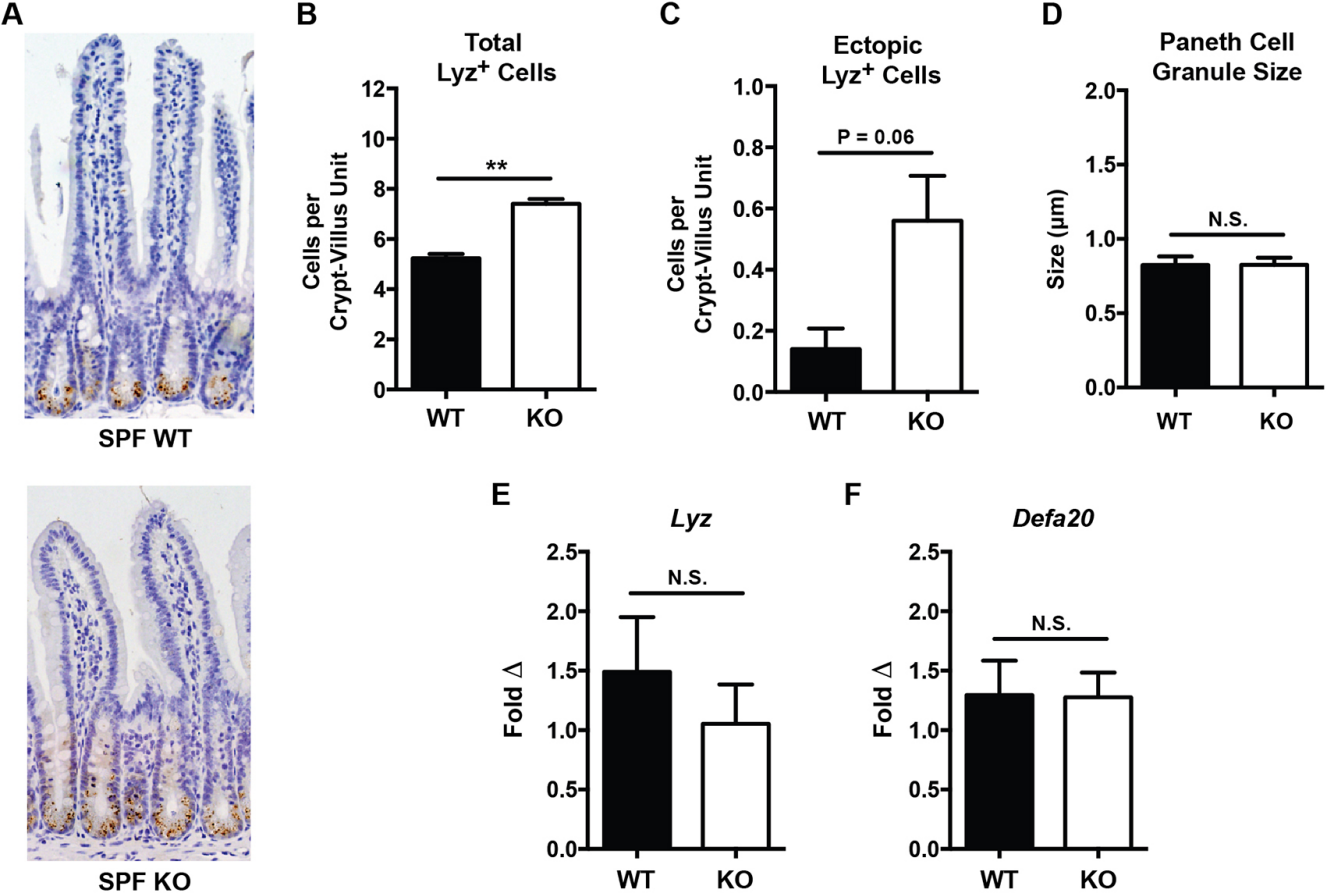
**Histologic and functional Paneth cell measurements of specific pathogen-free *Irgm1*^−/−^ and wild-type mice.** (A) Representative ileal tissue sections from male specific pathogen-free (SPF) wild-type (WT) and *Irgm1*^−/−^ (KO) mice (Lyz IHC, 20× magnification). (B) Number of Lyz^+^ cells per histologic crypt/villus unit. (C) Number of ectopic Lyz^+^ cells existing outside the base of the crypt per crypt/villus unit. (D) Average individual PC granule size. (E,F) Results of quantitative RT-PCR measurement of ileal tissue transcript levels of the antimicrobial peptides *Lyz* and *Defa20* normalized to *β-actin*. *n*=6-12 mice/group, **P<0.008; error bars indicate s.e.m.; N.S., not significant.

In addition to enteric inflammatory responses to DSS, SPF *Irgm1^−/−^* mice were also evaluated for the PC anomalies previously observed in CV-raised KO animals. Similar to CV *Irgm1^−/−^* mice, SPF KO mice exhibited an increased number of PCs per histologic crypt-villus unit as compared to their WT controls (Fig. 3A,B). We also observed a trend towards increased ectopic PCs in SPF KO mice relative to their WT counterparts (Fig. 3C), though this did not reach statistical significance as we previously reported in CV KO mice (Liu et al., 2013). Notably, *Irgm1^−/−^* mice housed in SPF conditions failed to show differences in PC granule size compared to WT animals (Fig. 3D), a difference that was apparent in CV mice. Finally, we also measured the expression of genes related to PC function (including the antimicrobial peptides *Lyz* and *Defa20*) that have previously shown to be decreased in CV *Irgm1^−/−^* mice. Again, SPF *Irgm1^−/−^* mice showed no differences in expression of either of these genes compared to WT littermates (Fig. 3E,F). In summary, the PC abnormalities originally observed in CV *Irgm1^−/−^* mice are attenuated in SPF conditions.

### *Irgm1*-deficient and wild-type littermate mice display no differences in goblet cell numbers or epithelial proliferation in SPF conditions

**Fig. 4.**
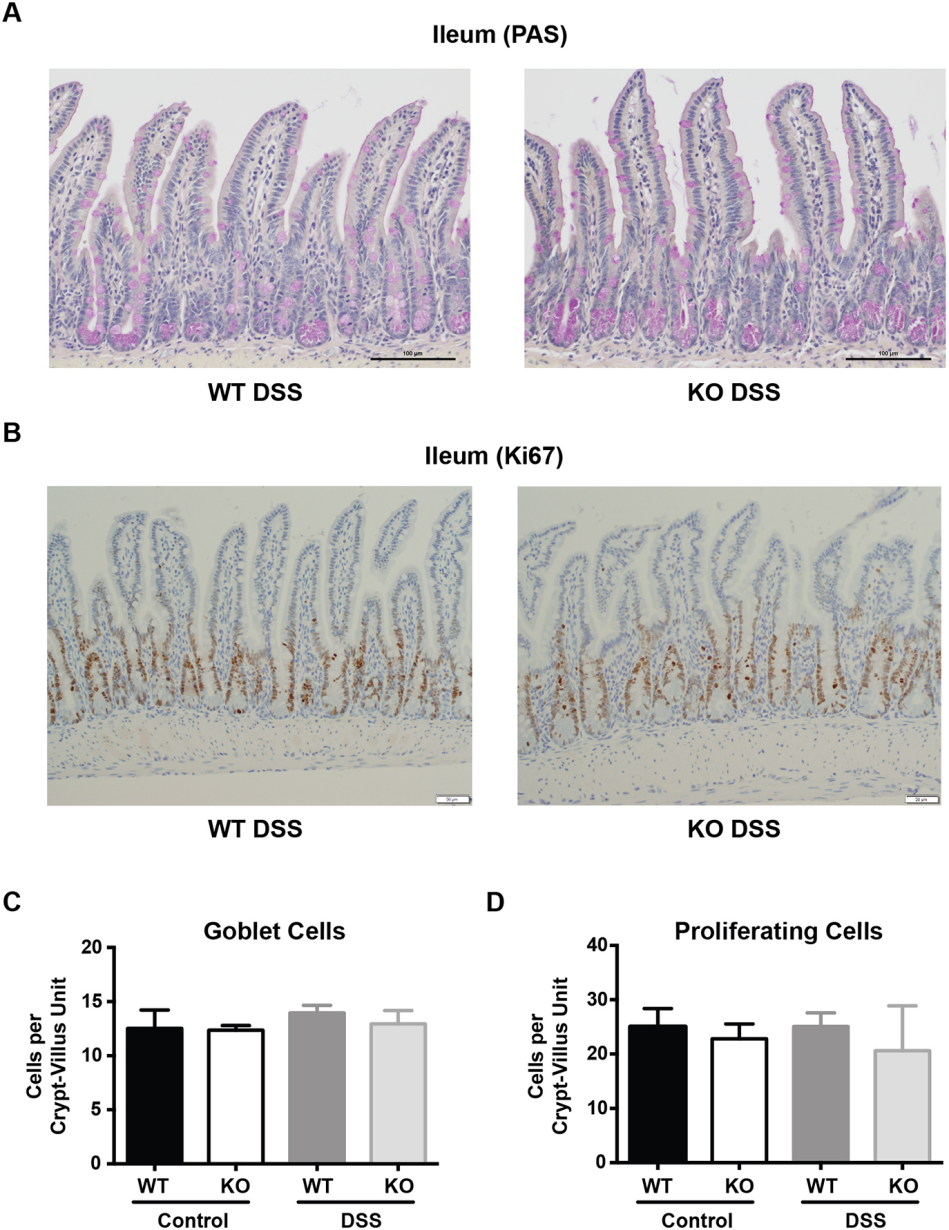
**Goblet cells and intestinal epithelial proliferation in specific pathogen-free *Irgm1*^−/−^ and wild-type mice.** Male specific pathogen-free (SPF) wild-type (WT) and *Irgm1*^−/−^ (KO) mice were administered 3% DSS in drinking water or drinking water alone (control) for 7 days. (A) Representative ileal tissue sections from DSS-treated SPF WT and KO mice (PAS, 20× magnification). (B) Representative ileal tissue sections from DSS-treated SPF WT and KO mice (Ki67 IHC, 20× magnification). (C) Number of goblet cells per crypt/villus unit, based on PAS staining. (D) Number of proliferating epithelial cells per crypt/villus unit, based on Ki67 staining. *n*=4-5 mice/group; error bars indicate s.e.m.

In addition to its role in PC biology, autophagy has also been implicated in the regulation of goblet cell function (Patel et al., 2013) and cellular proliferation (Sbrana et al., 2016). Therefore, we assessed goblet cell numbers and epithelial cell proliferation by using periodic acid–Schiff (PAS) and Ki67 staining, respectively, in the ileum of *Irgm1^−/−^* and WT littermates. As shown in Fig. 4A,C, goblet cell numbers were similar in WT and *Irgm1^−/−^* mice; however, we did observe an increased intensity of PAS staining in the goblet cells of KO animals. This was consistent across multiple sections from numerous mice. In regards to epithelial proliferation, we found no differences in the Ki67-positive zones of WT and *Irgm1^−/−^* mice (Fig. 4B,D). Importantly, there were also no ectopic foci of proliferation that would suggest aberrantly located crypt formation in the KO animals.

### Environment but not genotype drives microbial composition and diversity in *Irgm1*-deficient mice and wild-type littermates

**Fig. 5.**
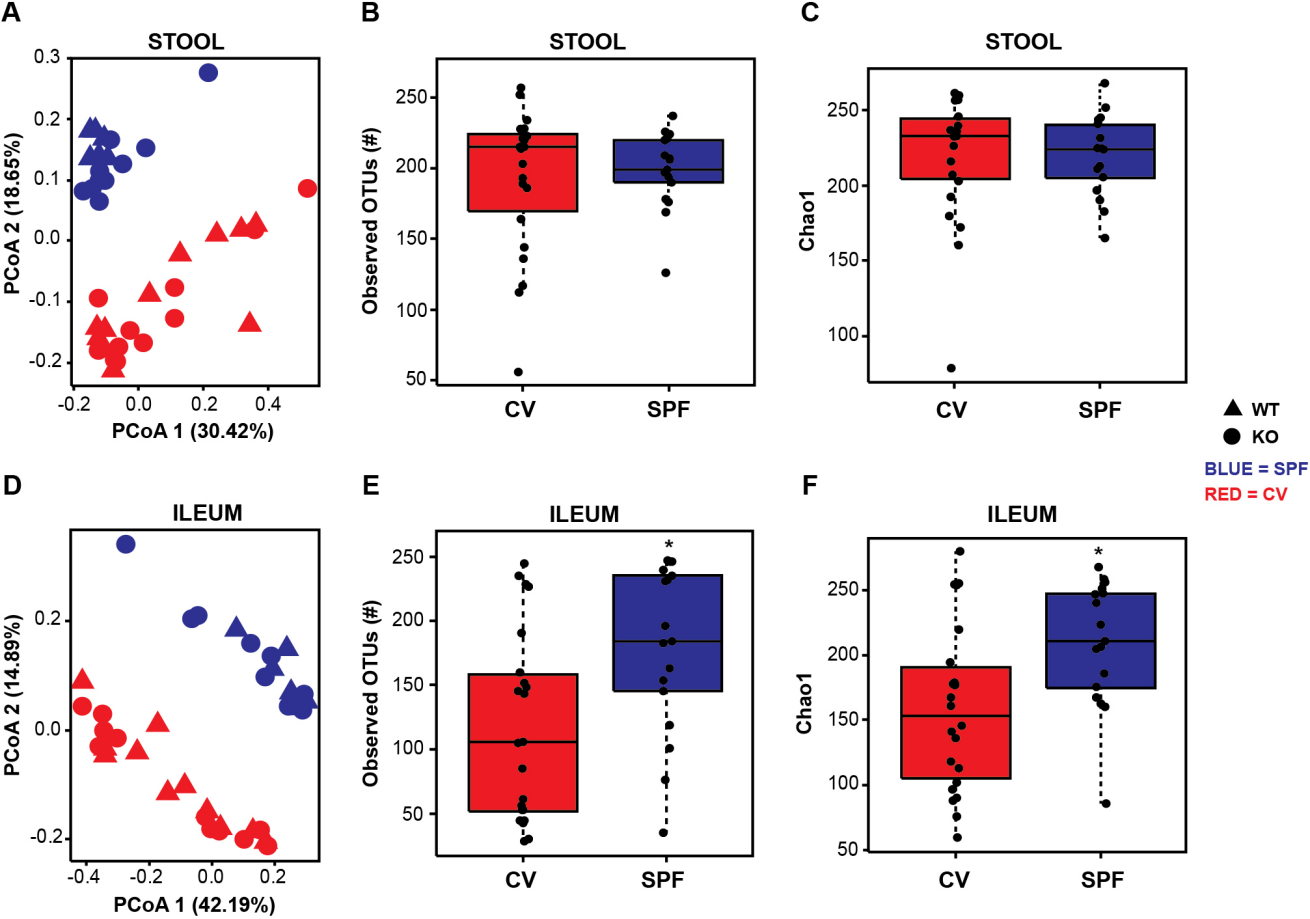
**Gut microbial composition and diversity differ based on housing conditions, but are not significantly influenced by genotype.** (A,D) Principal coordinates analysis of stool and ileal microbial composition of wild-type (WT) and *Irgm1^−/−^* (KO) mice in conventional (CV) and specific pathogen-free (SPF) conditions based on Bray–Curtis dissimilarity statistic. Richness measured using observed OTUs (B,E), and Chao1 index (C,F). *n*=11 CV WT, 11 CV KO, 7 SPF WT, 10 SPF KO. *FDR=0.024.

Given the differences in the inflammatory and PC phenotypes between populations of mice raised in different housing facilities, we next sought to determine whether the intestinal microbiota varied between groups. Deep sequencing of the V6 region of the 16S rRNA gene was performed on stool pellets and ileal tissue collected from *Irgm1* KO and WT mice reared in both housing conditions. Interestingly, principal coordinates analysis (PCoA) using Bray–Curtis dissimilarity showed no clustering of microbial communities based on *Irgm1* status; however, distinct differences between microbial communities from SPF and CV mice were observed in both stool and ileal tissue compartments (Fig. 5A,D). This is depicted statistically in Table 1. As indicated, the *Irgm1* genotype did not significantly impact microbial composition in stool or ileal samples for the first three PCoA axes, based on their *P*-values corrected for false discovery rate (FDR) (Table 1A). In contrast, housing conditions did significantly influence microbial composition for the first two PCoA axes in both stool and ileal compartments (Table 1B). Notably, similar results were found through a parallel analysis using the QIIME platform (http://qiime.org/) (Fig. S1A).

**Table 1.**
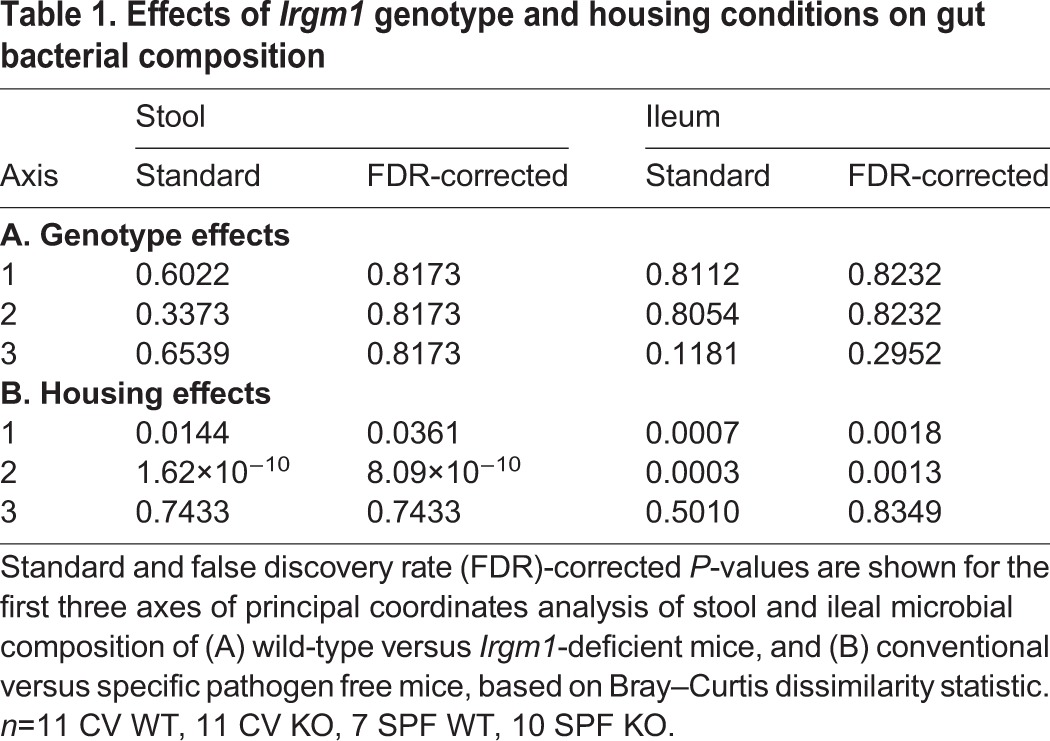
**Effects of *Irgm1* genotype and housing conditions on gut bacterial composition**

In addition to its effects on PCoA clustering (β-diversity), the housing environment also significantly impacted the richness of the ileal microbiota, as measured by observed operational taxonomic units (OTU) numbers (Fig. 5B,E) and the Chao1 index (Fig. 5C,F). Specifically, CV mice had a decreased richness of their ileal microbiota (FDR=0.024) when compared to SPF mice. The richness of the fecal microbiota between these two groups was similar (FDR>0.66). Again, *Irgm1* gene status did not appear to influence the richness of the ileal (FDR=0.817) or fecal (FDR=0.375) microbiota. Similar results were obtained using the QIIME platform (Fig. S1B,C). In total, these findings suggest that environmental factors, as opposed to *Irgm1* expression, are the primary drivers of gut microbial composition and diversity in these animals.

### Microbial influences modulate the PC morphology in *Irgm1*-deficient mice

**Fig. 6.**
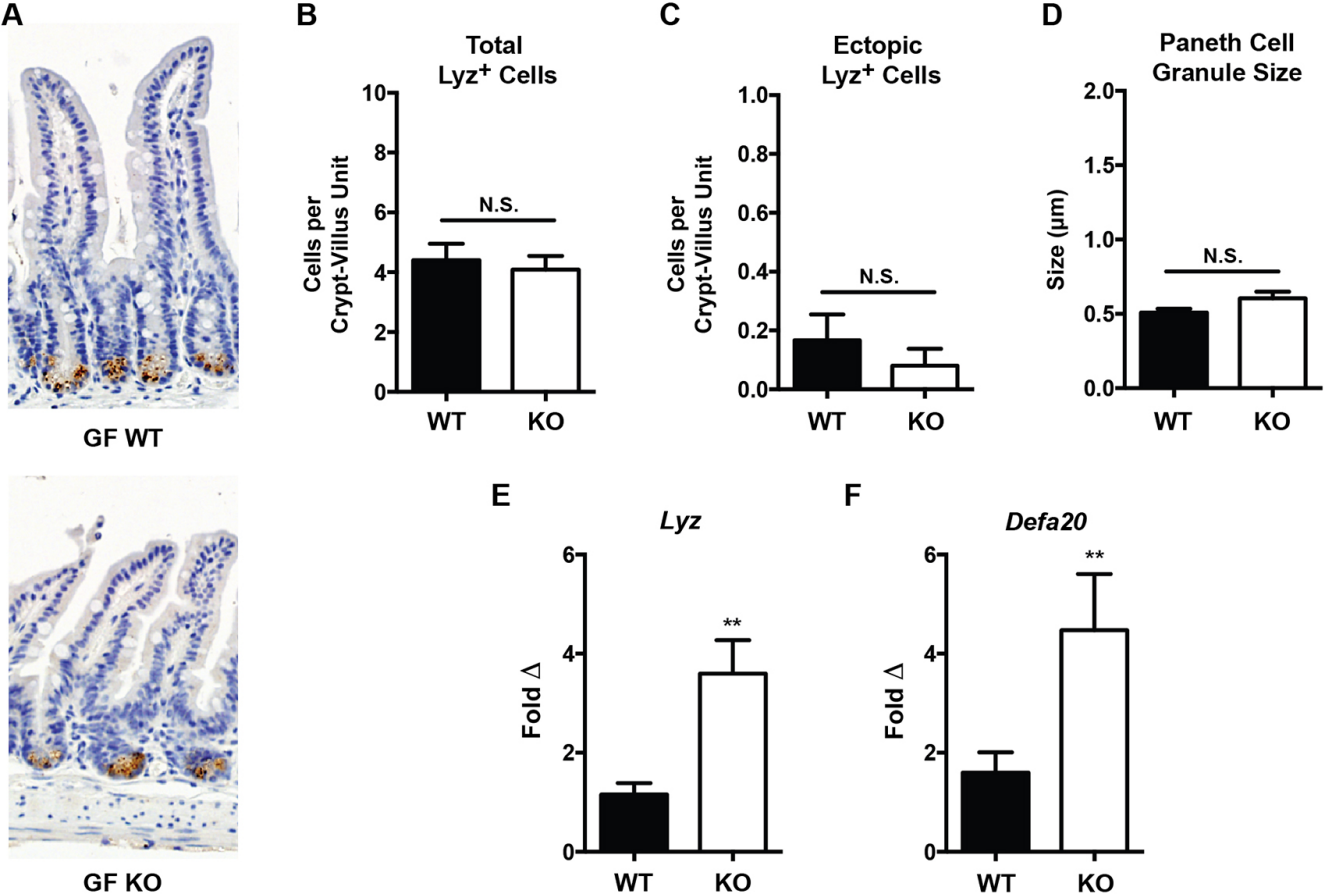
**Histologic and functional Paneth cell measurements of germ-free *Irgm1*^−/−^ and wild-type mice.** (A) Representative ileal tissue sections from germ-free (GF) wild-type (WT) and *Irgm1*^−/−^ (KO) mice (Lyz IHC, 20× magnification). (B) Number of Lyz^+^ cells per histologic crypt/villus unit. (C) Number of ectopic Lyz^+^ cells existing outside the base of the crypt per crypt/villus unit. (D) Average individual PC granule size. (E,F) Results of quantitative RT-PCR measurement of ileal tissue transcript levels of the antimicrobial peptides *Lyz* and *Defa20* normalized to *β-actin*. *n*=6-12 mice/group, **P<0.005; error bars indicate s.e.m.; N.S., not significant.

Given our finding that SPF and CV housing facilities support markedly distinct enteric bacterial communities in both KO and WT mice, we speculated that a microbial driver is responsible for the differences in PC phenotype we observed in these two environmental conditions. To test this hypothesis, we rederived our Irgm1 colony into GF conditions and assessed their PCs for both morphology and AMP expression. Similar to the SPF colony (but in contrast to CV-raised mice), there were no significant differences in PC number, location and granule size between WT and KO mice (Fig. 6A-D). Intriguingly, however, we found that GF KO mice showed increased mRNA expression of *Lyz* and *Defa20* compared to WT mice (Fig. 6E,F). These findings suggest that the gut microbiota influences PC morphology in *Irgm1*-deficient mice, though there appears be an effect of *Irgm1* itself on gene expression of AMPs.

### *H. hepaticus* is not sufficient to reconstitute the inflammatory phenotype of conventional *Irgm1*-deficient mice

**Fig. 7.**
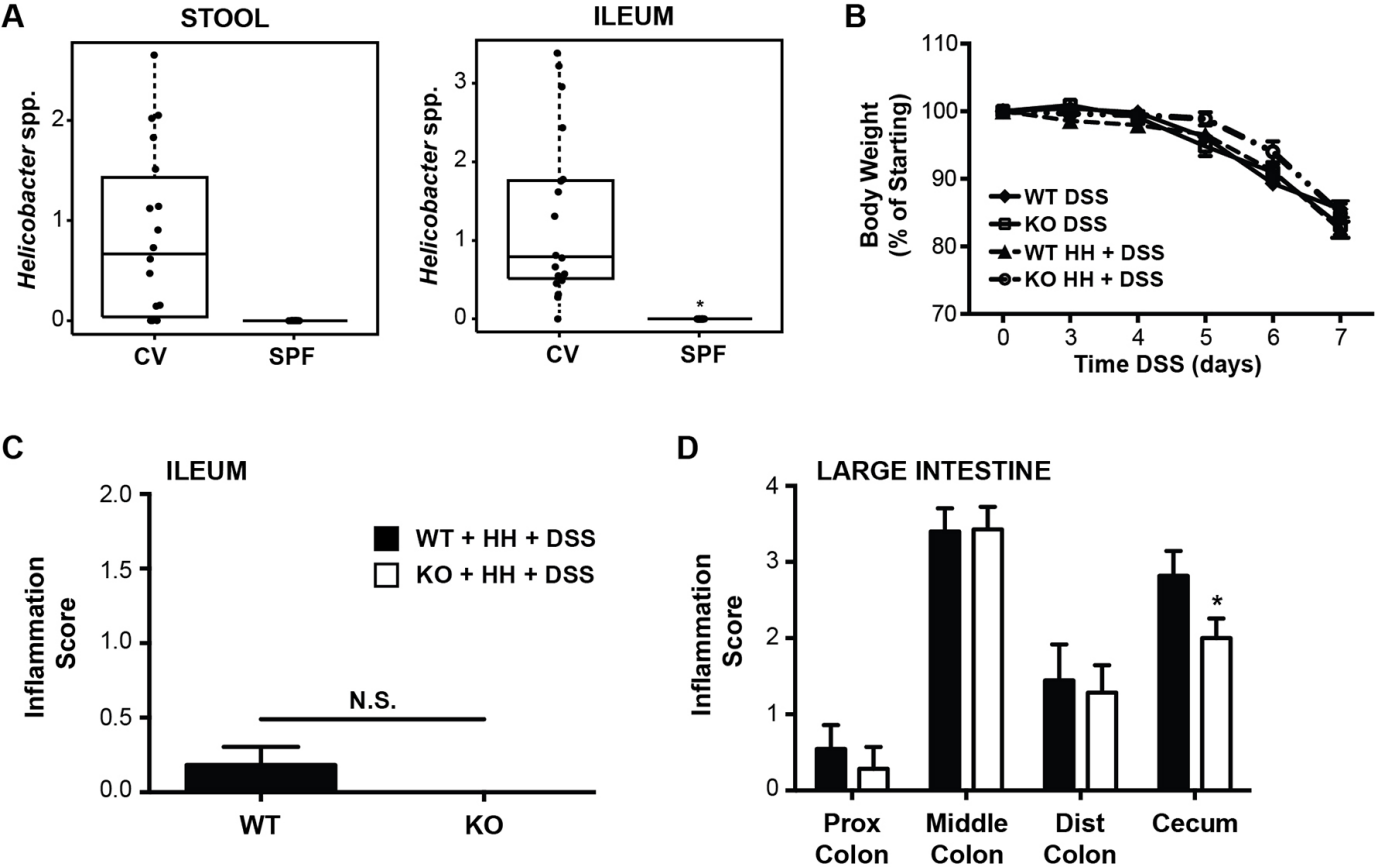
**Clinical and pathologic response of *H. hepaticus*-infected *Irgm1*^−/−^ mice to treatment with DSS.** (A) Relative abundance of Helicobacter species in stool and ileal samples of untreated conventional (CV) and specific pathogen-free (SPF) mice. *FDR<0.009 (ileum); FDR=0.125 (stool). *n*=11 CV wild-type (WT), 11 CV KO, 7 SPF WT, 10 SPF KO. For B-D, male SPF WT and KO mice were infected with *H. hepaticus* (HH) for 6 weeks, followed by administration of 3% DSS in drinking water for 7 days. (B) Weight loss shown as a percentage of initial weight prior to DSS treatment, *P*>0.1 (two-way ANOVA). (C-D) Average histologic inflammation scores in the ileum and large intestine. For B-D, values are means±s.e., combined from two separate studies. Cohort sizes were in the range of 7-14 mice/group, *P<0.05; error bars indicate s.e.m.; N.S., not significant.

The observed differences in gut-bacterial communities in CV versus SPF mice also allowed us to generate hypotheses regarding the impact of specific bacterial groups on DSS susceptibility in *Irgm1*-deficient animals. Specifically, we were able to determine which bacterial groups were increased in relative abundance in CV versus SPF housing conditions. A complete list of these organisms can be found in Table S1. One bacterial group of particular interest that was increased in the CV versus SPF samples was the *Helicobacter* genus (Fig. 7A). Because various *Helicobacter* spp. have been shown to enhance intestinal inflammation in numerous murine models of colitis (Chichlowski and Hale, 2009), we sought to determine whether this organism could be a driver of the inflammatory phenotype observed in our CV *Irgm1* KO mice.

Quantitative PCR testing for specific *Helicobacter* groups identified *H. hepaticus* as the most abundant species in the stool samples of CV mice. Therefore, we next assessed whether *H. hepaticus* colonization of SPF *Irgm1* KO mice would enhance their inflammatory response to treatment with DSS. To accomplish this, we colonized SPF *Irgm1* KO and WT mice with *H. hepaticus*, administered DSS, and then assessed for intestinal inflammation. In these studies, we found no differences in weight loss between DSS-treated *Irgm1* KO and WT mice that had been pre-infected with *H. hepaticus* (Fig. 7B). Moreover, all experimental groups developed similar histologic inflammation in both the ileum and the colon (Fig. 7C,D). Intriguingly, contrary to our original hypothesis, less inflammation was seen in the cecum of KO mice receiving *H. hepaticus*+DSS, as compared to similarly treated WT animals. Regardless, the introduction of *H. hepaticus* was not sufficient to recrudesce the inflammatory phenotype previously shown in CV KO mice.

## DISCUSSION

To date, genome-wide association studies (GWAS) have linked 206 genetic loci to the development of IBD (Ellinghaus et al., 2016). However, the cumulative disease risk described by these studies is ∼25% (Denson et al., 2013), a relatively small fraction of overall IBD heritability. Moreover, the concordance rates for IBD in homozygous twins is ∼50% for CD and ∼20% for ulcerative colitis (Halfvarson et al., 2003). These findings suggest that environmental influences play a key role in dictating the penetrance of IBD phenotypes. In this present study, we demonstrate that housing conditions modulate the susceptibility of *Irgm1*-deficient mice to intestinal injury. Housing environment also interacts with *Irgm1* to regulate PC morphology and function. These findings are essential knowledge for investigators using the *Irgm1^−/−^* mouse model and set the stage for further understanding the cross talk between the environment and *Irgm1* to regulate mucosal homeostasis.

Although numerous IBD susceptibility genes have been identified, the precise mechanisms by which these molecules enhance disease risk remain unclear. *In vitro* studies have shown that human IRGM regulates key molecular processes, including mitochondrial fission (Singh et al., 2010), autophagy (Chauhan et al., 2015) and the clearance of intracellular pathogens (Kim et al., 2012). Limited studies of murine Irgm1 demonstrate similar functions of this mouse homologue. Specifically, mouse Irgm1 localizes to mitochondria and regulates fission (Henry et al., 2014). Irgm1 is also a mediator of IFNγ-induced autophagy and promotes the clearance of intracellular bacteria in cultured cells. Despite this *in vitro* knowledge, however, our understanding of how IRGM (and Irgm1) regulate intestinal inflammation *in vivo* is unclear.

We previously reported that *Irgm1*-deficient mice raised in CV housing conditions develop extensive intestinal injury after DSS exposure, as compared to WT mice (Liu et al., 2013). Specifically, DSS-treated CV KO mice develop more-severe disease in the ileum, middle colon, distal colon and cecum relative to WT animals. In contrast, the present study demonstrates that ‘cleaner’, DSS-treated SPF KO mice only incur more significant inflammation in the distal colon. Importantly, there is no increased susceptibility to ileitis in SPF KO mice treated with DSS. These data suggest that an environmental component modulates DSS responses in *Irgm1*-deficient animals.

A potential mechanism by which the environment modulates DSS susceptibility is via the enteric microbiota. It is well established that environmental influences can affect the composition and function of the gut microbiota (Ivanov et al., 2008; Thoene-Reineke et al., 2014). Moreover, variations in the enteric microbiota can influence the severity of DSS colitis (Brinkman et al., 2013; Nagalingam et al., 2011). Therefore, we characterized gut bacterial communities in WT and KO mice in both CV and SPF housing conditions. Remarkably, we found no impact of *Irgm1* itself on global enteric microbial composition in either CV or SPF environments. This is intriguing in light of previous work suggesting IRGM can promote the clearance of intracellular bacteria, such as *Mycobacterium tuberculosis* (Singh et al., 2006). That said, it is unclear how defects in intracellular bacterial killing affect the composition of the luminal/mucosal commensal microbiota, which is the focus of the present study. Such defects may be more relevant to pathogenic organisms, such as *Listeria monocytogenes* (Collazo et al., 2001) and *Salmonella typhimurium* (Henry et al., 2007), which do result in increased disease and bacterial burdens in *Irgm1^−/−^* mice. Additionally, Irgm1 may be primarily involved in mediating inflammatory responses to enteric bacteria, rather than shaping the composition of those bacteria. Despite the lack of a genotype effect, our microbiota analyses did demonstrate the gut microbiota of mice reared in CV conditions differed profoundly from those in SPF housing. This supports the possibility that the enteric microbiota may influence the variable inflammatory phenotypes exhibited between CV and SPF KO mice in response to DSS.

A complete list of gut bacterial taxa exhibiting differential abundances in CV versus SPF mice is shown in Table S1. Of interest is the *Helicobacter* genus, which is increased in CV mice compared to SPF counterparts. Numerous *Helicobacter* species have been shown to induce intestinal inflammation in susceptible mice, the most common of which include *H. hepaticus* and *H. bilis* (Chichlowski and Hale, 2009). By using species-specific, targeted qPCR, we identified *H. hepaticus* as the primary *Helicobacter* species present in our CV housing facility. This organism is a known trigger of intestinal inflammation in murine IBD models, such as the *IL10*-deficient mouse (Kuhn et al., 1993; Kullberg et al., 1998). Based on these findings, we postulated that *H. hepaticus* infection is able to recapitulate an inflammation-susceptible phenotype in our SPF *Irgm1* KO animals.

In contrast to our hypothesis, however, we found that *H. hepaticus* inoculation was not sufficient to increase the susceptibility of SPF *Irgm1* KO mice to DSS-induced intestinal injury over their WT counterparts. There are numerous putative explanations for this finding. First, it is possible that *H. hepaticus* is not the sole organism responsible for the increased susceptibility of CV *Irgm1* KO mice to DSS injury. Additional microbes, perhaps in concert with *H. hepaticus,* might be required to drive the inflammatory phenotype of CV KO animals. Indeed, previous work has shown that the composition of the commensal microbiota can regulate host inflammatory pathways that drive intestinal disease in *H. hepaticus* models of colitis (Nagalingam et al., 2013). A second possible explanation for these findings is that the strain of *H. hepaticus* used in our study might not be identical to that found in the CV animals. Ample evidence exists supporting the concept that different bacterial strains within a given species differ in their ability to induce colitis in animal models of IBD (Moran et al., 2009). However, it should be noted that the specific *H. hepaticus* strain used in this study (MU-94) has been shown to induce intestinal inflammation in susceptible mouse strains (Cook et al., 2014; Livingston et al., 2004). Finally, it also possible that the increased susceptibility to intestinal inflammation observed in CV *Irgm1* KO mice is not due to a transmissible microbial factor. Numerous environmental factors have been implicated in IBD pathogenesis (i.e. smoking, nonsteroidal anti-inflammatory drugs, appendectomy, diet, stress), and it is not clear whether these exert an impact on intestinal inflammation via the gut microbiota (Frolkis et al., 2013). Similarly, our housing facilities varied in regards to food, water, bedding and caging (Table S2). While it is likely that these differences may contribute to microbial variations between the colonies, it is also possible that they may impact host inflammatory responses through a microbial-independent mechanism.

In addition to its impact on inflammation susceptibility, housing environment also appears to interact with *Irgm1* to influence PC morphology and AMP transcription. We have shown that, in CV housing, *Irgm1*-deficient mice display multiple PC abnormalities including increased PC numbers, ectopic PCs, decreased PC granule size and decreased transcript levels of specific AMPs (*Lyz* and *Defa20*) (Liu et al., 2013). In the present study, *Irgm1* KO mice housed in SPF conditions continued to demonstrate increased PC numbers per crypt and a trend towards increased ectopic PCs. However, their granule morphology was identical to that of WT mice. Moreover, differences in *Lyz* and *Defa20* mRNA expression between WT and KO mice were abrogated in SPF conditions. Given the differences in bacterial communities between our CV and SPF facilities, these data suggest that the gut microbiota modulate PC function in *Irgm1*-deficient mice.

In order to dissect the relative contributions of genotype (*Irgm1* status) and the microbiota to PC regulation in this model, we next rederived *Irgm1*-deficient mice and WT littermates in GF housing conditions. Elimination of the microbiota resulted in complete loss of the morphological PC differences between WT and KO mice. Specifically, GF WT and KO mice displayed similar PC numbers, location and granule morphology. Intriguingly, transcript expression of *Lyz* and *Defa20* were increased in GF KO mice compared to their WT counterparts. Historically, these AMPs were not thought to be transcriptionally induced (Bevins and Salzman, 2011). For the α-defensins, however, elegant work by Wehkamp and colleagues has demonstrated transcriptional regulation of these molecules through Wnt signaling [via Tcf-1 (Beisner et al., 2014) and Tcf-4 (Wehkamp et al., 2007)]. Indeed, polymorphisms of the TCF-4 promoter have been associated with ileal CD (Koslowski et al., 2009). It is possible that the Wnt/β-catenin/Tcf pathway is upregulated in Irgm1-deficient mice, and future experiments are planned to explore this possibility. Presently, however, we can surmise that these findings contrast with the decreased *Defa20* and *Lyz* mRNA expression observed in CV KO mice, and the equivalent AMP expression shown between SPF KO mice and their WT counterparts. This suggests that the gut microbiota influences the manner in which *Irgm1* regulates AMP expression.

To date, the precise microbial constituents that regulate PC phenotype in the *Irgm1*-deficient mouse model remain unclear. Previous work, studying the autophagy-related CD risk allele ATG16L1, has demonstrated that Atg16l1 hypomorph mice have a similar PC phenotype to our CV *Irgm1* KO animals. Specifically, Atg16l1^HM^ mice possess dysmorphic PCs with reduced numbers of lysozyme granules (Cadwell et al., 2008, 2009). Remarkably, this abnormal PC phenotype is dependent on a distinct murine norovirus strain (MNV-CR6) (Cadwell et al., 2010). Notably, murine norovirus is excluded from our SPF mouse colony and, hence, SPF *Irgm1* KO mice are not exposed to this microbe (Table S3). Future studies will attempt to recapitulate a dysmorphic PC phenotype in *Irgm1* KO mice by colonizing with the MNV-CR6 strain. Intriguingly, we did observe subtle differences in the ileal goblet cells of SPF *Irgm1^−/−^* mice. Although goblet cell numbers were similar between WT and KO mice, the increased intensity of PAS staining in KO animals could indicate differences in the mucin composition of these cells. This will also be explored further in future studies.

The findings presented in this study demonstrate that environmental conditions interact with *Irgm1*, putatively through the enteric microbiota, to direct PC function and host-susceptibility to intestinal injury. This might be a critical consequence of the role Irgm1 plays in regulating autophagy. An established function of autophagy is to assist in the clearance of certain intracellular organisms (Levine et al., 2011). This antimicrobial process, known as xenophagy, can be triggered by pathogen-associated molecular patterns (Deretic et al., 2015). As such, a mechanism is required to transmit microbial inputs to the initiation of xenophagic processes. Recently, human IRGM has been shown to directly interact with the pattern-recognition receptor NOD2, forming a core complex that can regulate autophagy in response to microbial products (Chauhan et al., 2015). The interaction between the environment and *Irgm1* demonstrated in the present study may support a similar role for this murine homologue. It is possible that *Irgm1* serves to integrate environmental inputs, such as the microbiota, into signals that can regulate autophagy within host cells. While a comprehensive evaluation of the impact of environment on autophagy in *Irgm1*-deficient mice is beyond the scope of the present study, future work will be directed at dissecting the mechanistic aspects of this putative *Irgm1* function.

Overall, the findings described in this study highlight the importance of the *Irgm1*-deficient mouse model for the study of human CD. The pathogenesis of CD is multifactorial, involving both environmental and host factors that interact to drive disease in a susceptible individual. *Irgm1* KO mice appear to display a similar phenotype, in which environmental influences are able to modulate the impact of *Irgm1* deficiency on PC morphology and inflammation susceptibility. The findings presented also parallel work in human CD, which has demonstrated that abnormal PC morphology in ileal biopsies may predict a more-rapid time to recurrence in patients with CD (VanDussen et al., 2014). Although direct causality between dysmorphic PCs and susceptibility to DSS has not been shown in the *Irgm1* KO model, a similar phenomenon is observed in these mice: CV KO mice with abnormal PCs are more susceptible to DSS-mediated injury, while SPF KO mice have relatively normal PCs and less-severe responses to DSS.

In summary, our present study characterizes novel aspects of the *Irgm1^−/−^* mouse model that are important to the scientific community and are highly relevant to the understanding of human CD. In particular, environmental conditions strongly impact the influence of Irgm1 on PC and inflammatory phenotypes in the mouse small intestine. In contrast, Irgm1 itself does not appear to regulate gut microbial composition. Future work using this model may now focus on how, mechanistically, abnormal PCs drive this increased susceptibility to intestinal injury. Importantly, the role of environmental factors in this process may also be elucidated. Characterizing these gene-environment interactions will enhance our understanding of the role of IRGM in human CD and, ultimately, promote the development of novel preventative and therapeutic approaches for these patients.

## MATERIALS AND METHODS

### Mice

All animals used in this study were *Mus musculus*, C57BL/6, sex-matched, adult (2-4 months old) mice. SPF and GF *Irgm1* heterozygous mice were rederived from mice housed in CV conditions (Liu et al., 2013; Collazo et al., 2001), by implantation of two-cell stage embryos into WT SPF or GF pseudopregnant females. Heterozygous mice were then mated to produce *Irgm1* KO and WT littermate animals for experimental use. Husbandry details for each facility are provided in Table S2. Importantly, our SPF facility excludes *Helicobacter* spp. and murine norovirus. A complete list of organisms excluded in our CV and SPF facilities is shown in Table S3. All mice were housed under AAALAC-accredited facilities and IACUC-approved protocols at the Durham VA Medical Center, Duke University Medical Center, and the University of North Carolina Medical Center, respectively.

### DSS-induced intestinal injury

Acute intestinal injury was induced as previously described (Liu et al., 2013). Briefly, male mice received 3% (w/v) dextran sodium sulfate (DSS) (MP Biomedicals, #160110) in their drinking water for 7 days. Control mice received drinking water without DSS. Mice were weighed and feces collected on days 0, 3, 5, 6 and 7. Fecal blood and stool consistency were assessed at each time point as previously described (Liu et al., 2013). Mice were killed on day 7, and their intestinal tissues collected for analysis. Colons were removed and length measured prior to being opened longitudinally and content removed. Colons were then rolled in a Swiss-roll fashion. Ceca were also opened longitudinally, their content removed and rolled for histology. Ileal tissue was opened longitudinally, gross contents were removed and the remaining tissue fixed flat prior to paraffin embedding. Tissues bound for histologic analysis were fixed in 10% formalin solution for 48 h prior to paraffin embedding. Sections were cut (5 µm) and stained with hematoxylin and eosin (H&E). Histologic inflammation was scored in a blinded fashion using validated systems for ileum (Moran et al., 2009) and DSS-induced colitis (Erben et al., 2014).

### Immunohistochemistry

Immunohistochemistry (IHC) was performed for Ki67 and Lyz using 5 µm, formalin-fixed, paraffin-embedded ileal tissue sections as described above. De-paraffinization was accomplished by using fresh xylene, followed by re-hydration via a series of ethanol dilutions. Heat-induced epitope retrieval was subsequently utilized for antigen retrieval (ThermoFisher, #TA-135-HBL). This was followed by treatment with 3% hydrogen peroxide to quench endogenous peroxidase activity. Blocking was performed with 10% normal goat serum. After sections were prepared in this fashion, they were incubated overnight at 4°C with either anti-Ki67 (1:250, Vector Laboratories, #VP-RM04) or anti-Lyz (1:100, Diagnostic Biosystems, #RP028) rabbit primary antibodies. Sections were next incubated for 1 h at room temperature with a biotinylated goat anti-rabbit IgG secondary antibody (1:500, Jackson ImmunoResearch, #111-065-144). Signal amplification was accomplished using the Vectastain Elite ABC-HRP Kit per manufacturer's instructions (1:50, Vector Laboratories, #PK-6100). Finally, staining was visualized with DAB chromoreagent (ThermoFisher, #TA-125-QHDX).

### Epithelial cell analyses

Epithelial cell subtypes were enumerated in a blinded fashion and reported as number of cells per crypt-villus unit. To be included in the analysis, a crypt-villus unit was required to contain a properly oriented crypt (full crypt with lumen visible in cross section) and visibly adjacent villi. At least 10 crypt-villus units were evaluated for each mouse. Paneth cells (PCs) were enumerated by counting the number of Lyz IHC positive cells within a histologic crypt-villus unit. Ectopic PCs were recorded as the number of PCs identified outside their normal position within the crypt base. PC granule sizes were measured on H&E stained sections, using the ellipse tool in ImageJ version 1.48 software (NIH). At least 70 granules from varying regions were measured for each mouse. Goblet cells were enumerated per crypt-villus unit using periodic-acid Schiff (PAS) staining (ThermoFisher, #SS32-500), which readily identifies deep purple cells with classic goblet cell morphology. Finally, proliferating epithelial cells were assessed by counting the number of Ki67 IHC positive cells per crypt-villus unit.

### Quantitative RT-PCR

Ileal tissues were collected into RNA*later* solution (Qiagen, #76104) for subsequent RNA isolation. Total RNA was extracted from flushed ileal tissue using an RNeasy Mini Kit per manufacturer's instructions (Qiagen, #74104). Complementary DNA was generated using SuperScript II Reverse Transcriptase (Invitrogen, #18064014). Quantitative reverse-transcriptase polymerase chain reaction (RT-PCR) was conducted for *Lyz* (Applied Biosystems, Mm00657323_m1) and *Defa20* (Applied Biosystems, Mm00842045_g1). These specific AMPs were chosen because they have previously been shown to differ between *Irgm1* KO and WT mice (Liu et al., 2013). Each sample was run in triplicate via quantitative RT-PCR using TaqMan Gene Expression Master Mix and the appropriate primer/probe set for the gene of interest (ThermoFisher Scientific, #4369016). Results were analyzed by using the comparative threshold cycle (ΔΔCt) method, normalized against β-actin (Applied Biosystems, Mm02619580_g1) and compared with the baseline WT group.

### Deep sequencing of gut-bacterial communities

Bacterial DNA was extracted from 100 mg of frozen tissue or feces as previously described (Shanahan et al., 2014). Briefly, samples were incubated in a sterile lysis buffer [200 mM NaCl, 100 mM Tris pH 8.0, 20 mM EDTA, 20 mg/ml lysozyme (Sigma-Aldrich, #12671-19-1)] for 30 min at 37°C. Lysis was completed by addition of 40 µl of proteinase K (20 mg/ml) and 85 µl of 10% SDS to the mixture, and incubation for an additional 30 min at 65°C. Samples were next homogenized by bead beating for 2 min using 300 mg/sample of 0.1 mm zirconium beads (BioSpec Products, #11079101Z). DNA was extracted from the resultant supernatants using phenol/chloroform/isoamyl alcohol (25:24:1), and precipitated with absolute ethanol for 1 h at −20°C. Precipitated DNA was purified by using a DNeasy Blood and Tissue extraction kit (Qiagen, #69504) per manufacturer's instructions.

The purified DNA samples were used to construct an Illumina V6 16S rRNA library for deep sequencing, as previously described (Arthur et al., 2012). Briefly, an initial PCR was performed using barcoded universal V6 primers (Table S3). This allowed for amplification of the V6 region, with simultaneous addition of unique barcodes for multiplex analyses. 15 μl of this PCR product was then used in a second PCR reaction using PCRF1/PCRR1 primers (Table S4), to add Illumina flow cell adaptor sequences to the final product. The final PCR amplicons were purified using a Qiagen PCR Purification Kit and visualized on a 1.5% agarose gel to confirm size and purity. Finally, equal amounts of DNA from each sample were pooled (final concentration 21 ng/μl) and subject to 100 bp paired-end read sequencing, using the Illumina HiSeq2000 platform.

### Analysis of 16S rRNA sequences

A total of 88,015,334 paired-end reads were generated and processed as described previously (Arthur et al., 2014). Forward and reverse reads were merged using a minimum of 70 continuous matching bases between them, which resulted in 48,999,102 merged sequences for the current study, representing 78 samples. We used AbundantOTU+ v.0.93b (http://omics.informatics.indiana.edu/AbundantOTU/out+.php) with the ‘-abundantonly’ option to cluster these sequences into 1468 Operational Taxonomic Units (OTUs), incorporating 99.86% of the input sequences. UCHIME (http://www.drive5.com/uchime/) (Edgar et al., 2011) and the Gold reference database were used to screen for the presence of chimeras in our OTU sequences, and a total of eight OTUs were removed. The remaining 1460 OTUs were used for downstream analysis. Taxonomic assignments were done using uclust through QIIME assign_taxonomy.py (Caporaso et al., 2010). A parallel analysis using QIIME v.1.9.1 was also conducted, utilizing the close-reference OTU picking approach (at 97% similarity level using the Greengenes 97% reference dataset, release 13_8). We excluded OTUs that had ≤0.005% of the total number of sequences according to Bokulich and colleagues (Bokulich et al., 2013).

PCoA plots were generated from both Bray–Curtis dissimilarity statistic on the normalized and log_10_ transformed reads (Arthur et al., 2014), and UniFrac after rarefying the counts to the minimum number of reads found in all samples (32,698 for Ileum and 78,957 for stool samples). Alpha diversity (observed OTU estimate and Chao1 diversity index) was calculated after rarefying the raw counts to a depth of the minimum count in all samples (32,698 for Ileum and 78,957 for stool samples). We used a linear mixed-effects model utilizing the package nlme_v. 3.1-128 in R (v. 3.3.1) (http://www.R-project.org/) to analyze the data and account for possible contributions that may arise from co-housing the mice in the same cage. In our model, cage was modeled as a random effect and both *Irgm1* status and housing condition as fixed effects (McCafferty et al., 2013). *P*-values are reported from the linear mixed-effects model using *F*-test. We controlled for false discovery rate (FDR) by correcting the *P*-values using the Benjamini and Hochberg (BH) approach (Benjamini et al., 2001).

### *Helicobacter* studies

Testing of fecal matter for *Helicobacter* colonization was conducted via commercial PCR assays utilizing *Helicobacter* genus-level primers, followed by speciation using species-specific primers (IDEXX BioResearch, Columbia, MO). For *H. hepaticus* colonization experiments, the MU-94 strain was obtained as a kind gift from Dr Robert Livingstone (IDEXX BioResearch). SPF mice received 1×10^8^ colony forming units (CFU) of *H. hepaticus* suspended in 200 μl of Brucella broth that was gavaged on three separate occasions spaced 3-4 days apart (Livingston et al., 2004). After each gavage dosing, an additional 1 ml of *H. hepaticus*-infected broth was added to the floor of each cage. *Helicobacter* colonization using this protocol was confirmed by PCR. Mice were held for 6 weeks before being exposed to a 7-day DSS treatment and then killed as described above.

### Statistical analysis

All statistical analyses, other than sequencing analysis described above, were performed using Graphpad Prism version 6 (Graphpad software Inc., San Diego, CA). Results are expressed as standard error of the mean (s.e.m.). Clinical data were analyzed using two-way analysis of variance (ANOVA) with other comparisons analyzed using the Kruskal–Wallis and Mann–Whitney tests. A *P*<0.05 was considered significant.

## Supplementary Material

Supplementary information
