# Supplementary material for: Environmental factors regulate Paneth cell phenotype and host susceptibility to intestinal inflammation in Irgm1-deficient mice
Source: Dis Model Mech. 2018 Feb 1;11(2):dmm031070. doi: 10.1242/dmm.031070 (PMC5894938; doi:10.1242/dmm.031070)
Supplement: Supplementary information [file dmm-11-031070-s1.pdf]

SUPPLEMENTAL MATERIAL

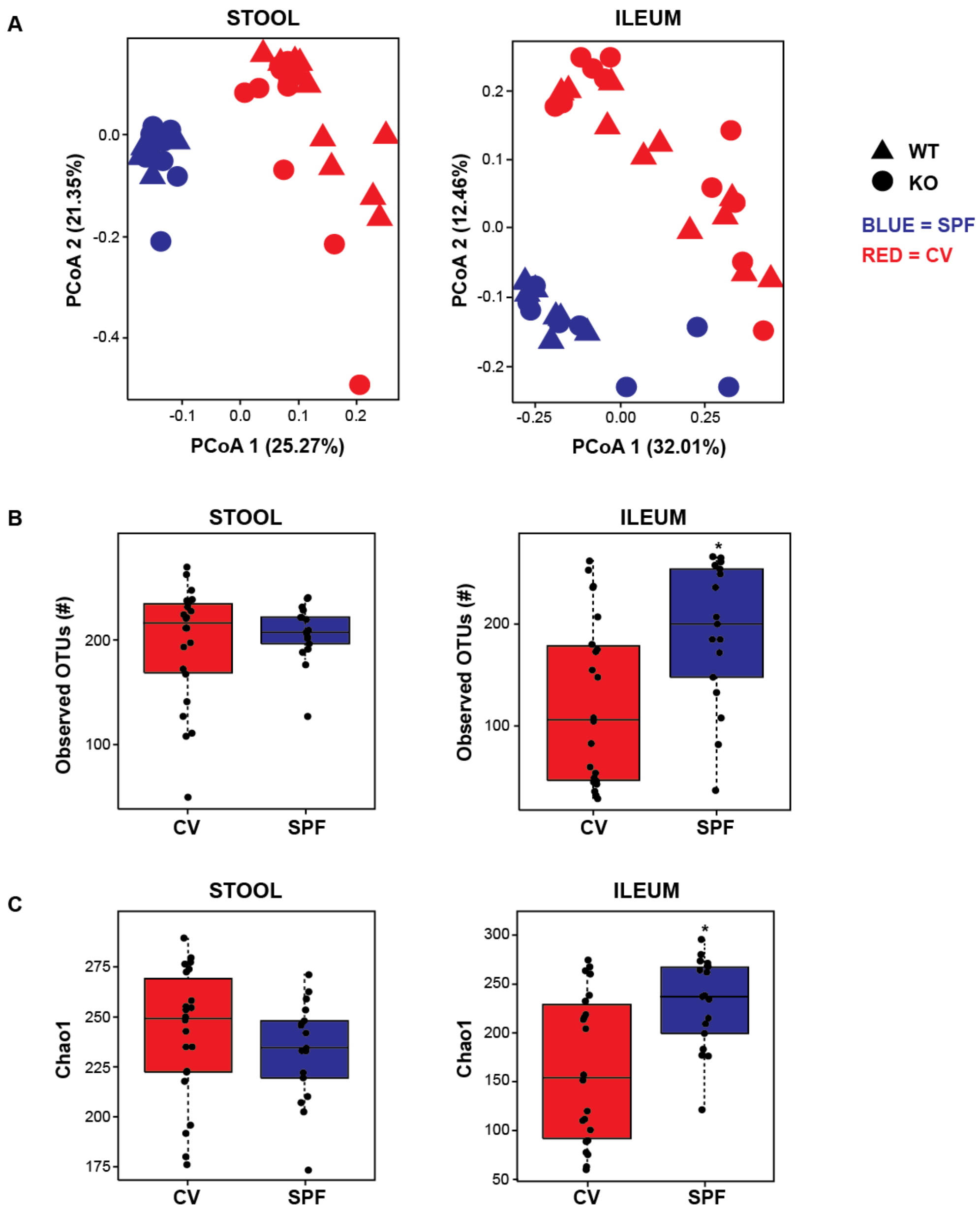

Fig. S1. *QIIME analysis confirms gut microbial composition and diversity differ between housing conditions, but not genotype.* A: Principal coordinates analysis of stool and ileal microbial composition of WT and *Irgm1* KO mice in conventional (CV) and specific pathogen free (SPF) conditions based on QIIME UniFrac distances. Stool CV versus SPF, FDR < $6.4x10^{-10}$; ileum CV versus SPF, FDR < 0.007. For WT versus KO mice in both tissue compartments, FDR > 0.09. B: Observed OTUs calculated from QIIME close reference

Disease Models & Mechanisms • Supplementary information

pipeline. C: Chao1 α-diversity index calculated from QIIME close reference pipeline. N = 11 CV WT, 11 CV KO, 7 SPF WT, 10 SPF KO. *FDR = 0.021.

Disease Models & Mechanisms • Supplementary information

Supplemental Tables

Genus	Compartment	P-value	FDR
Akkermansia	Stool	2.3617E-06	8.26593E-05
Bifidobacterium	Stool	7.89538E-06	0.000138169
Butyricimonas	Stool	0.000466406	0.004081048
Dorea	Stool	0.000461691	0.004081048
Anaeroplasma	Stool	0.001140313	0.007982192
Vagococcus	Ileum	1.38967E-12	7.22626E-11
Chelonobacter	Ileum	8.79941E-12	2.28785E-10
Erysipelothrix	Ileum	2.20262E-11	3.81787E-10
Pseudochrobactrum	Ileum	8.97944E-11	1.16733E-09
Marinomonas	Ileum	3.59938E-10	3.74336E-09
Halomonas	Ileum	5.11173E-10	4.43016E-09
Proteiniclasticum	Ileum	8.98642E-10	6.67563E-09
Leucobacter	Ileum	5.99842E-09	3.89897E-08
Agrococcus	Ileum	7.33039E-09	4.23534E-08
Dysgonomonas	Ileum	8.6816E-09	4.51443E-08
Psychrobacter	Ileum	2.63154E-07	1.244E-06
Rhodococcus	Ileum	7.73384E-07	3.35133E-06
Shewanella	Ileum	1.07208E-06	4.28834E-06
Aquimarina	Ileum	2.24452E-06	8.33678E-06
Bifidobacterium	Ileum	1.22284E-05	4.23919E-05
Anaeroplasma	Ileum	1.34341E-05	4.36609E-05
Chryseobacterium	Ileum	2.31769E-05	7.08939E-05
Dorea	Ileum	2.89641E-05	8.3674E-05
Akkermansia	Ileum	0.000197202	0.000539711
Microbacterium	Ileum	0.000295184	0.000767478
Coprobacillus	Ileum	0.000337282	0.000835173
Blautia	Ileum	0.000587913	0.001329194
Clostridium	Ileum	0.000568876	0.001329194
Trichococcus	Ileum	0.001315745	0.002850781

Disease Models & Mechanisms • Supplementary information

Butyricimonas	Ileum	0.001819406	0.003784364
Vibrio	Ileum	0.002193962	0.004387924
Helicobacter	Ileum	0.004657984	0.008970933
Enterococcus	Ileum	0.007563428	0.014046366
Adlercreutzia	Ileum	0.013553457	0.02430275
Oscillospira	Ileum	0.016514794	0.028625644
Desulfovibrio	Ileum	0.022523324	0.036600401
Staphylococcus	Ileum	0.022233868	0.036600401
AF12	Ileum	0.028106572	0.044289144
Pseudomonas	Ileum	0.030143274	0.046101478
Flavobacterium	Ileum	0.03219191	0.047827981
Acinetobacter	Ileum	0.033112258	0.047828818

Table S1. *Bacterial taxa differentially abundant in conventional (CV) versus specific pathogen free (SPF) facilities.* Genus-level bacterial taxa that are significantly different in CV versus SPF facilities are shown. *P*-values are reported from the mixed linear model using *F*-test, which accounts for the contribution of cage. We controlled for false discovery rate (FDR) by correcting the *P*-values using Benjamini and Hochberg (BH) approach.

Disease Models & Mechanisms • Supplementary information

	CV	SPF	GF
**Feed**	Purina 5001	Purina 5053 (irradiated)	Teklad Global 2020SX (autoclaved)
**Water**	Triple filtered	Reverse osmosis	Autoclaved
**Bedding**	ALPHA-dri/Cob blend	Corncob (autoclaved)	Teklad 7070C Diamond Dry Cellulose (autoclaved)
**Caging**	Individual ventilated cages (hot-washed)	Shoebox cages (autoclaved)	Shoebox cages (autoclaved) within flexible film isolators

Table S2. *Husbandry details for individual mouse facilities.* Specific food, water, bedding, enrichment, and caging sources are indicated for conventional (CV), specific pathogen free (SPF), and germ-free (GF) housing facilities. Sterilization techniques are also shown when utilized.

Disease Models & Mechanisms • Supplementary information

		**Conventional (CV)**	**Specific Pathogen Free (SPF)**
**Viral Agents**	Parvoviruses (MPV-1 & 2, Minute virus)	-	-
	Sendai virus	-	-
	Pneumonia virus of mice	-	-
	Mouse hepatitis virus	-	-
	Mouse norovirus	**+**	-
	Reovirus	-	-
	Enzootic diarrhea of mice	-	-
	Theiler's murine encephalomyelitis	-	-
**Bacterial Agents**	*Mycoplasma pulmonis*	-	-
	Helicobacter spp.	+	-
	Pasturella pneumotropica	Not tested	-

Table S3. *Colony health surveillance results from conventional (CV) and specific pathogen free (SPF) facilities.* Results of dirty bedding sentinel testing of either exposure (serology) or presence (PCR) of murine pathogens in the CV and SPF colonies during the time course of the study.

Disease Models & Mechanisms • Supplementary information

**V6F1**	5′-	ACACTCTTTCCCTACACGACGCTCTTCCGATCTATAGCGCAACGCGARGAACCTTACC	-3′
**V6F2**	5′-	ACACTCTTTCCCTACACGACGCTCTTCCGATCTAGGGTCAACGCGARGAACCTTACC	-3′
**V6F3**	5′-	ACACTCTTTCCCTACACGACGCTCTTCCGATCTTTCATCAACGCGARGAACCTTACC	-3′
**V6F4**	5′-	ACACTCTTTCCCTACACGACGCTCTTCCGATCTGATCGTCAACGCGARGAACCTTACC	-3′
**V6F5**	5′-	ACACTCTTTCCCTACACGACGCTCTTCCGATCTGCCCGTCAACGCGARGAACCTTACC	-3′
**V6F6**	5′-	ACACTCTTTCCCTACACGACGCTCTTCCGATCTCTGTCCAACGCGARGAACCTTACC	-3′
**V6F7**	5′-	ACACTCTTTCCCTACACGACGCTCTTCCGATCTCACGTCAACGCGARGAACCTTACC	-3′
**V6F8**	5′-	ACACTCTTTCCCTACACGACGCTCTTCCGATCTCGTACGCAACGCGARGAACCTTACC	-3′
**V6F9**	5′-	ACACTCTTTCCCTACACGACGCTCTTCCGATCTGGACCAACGCGARGAACCTTACC	-3′
**V6F10**	5′-	ACACTCTTTCCCTACACGACGCTCTTCCGATCTTAGACAACGCGARGAACCTTACC	-3′
**V6F11**	5′-	ACACTCTTTCCCTACACGACGCTCTTCCGATCTTCATCAACGCGARGAACCTTACC	-3′
**V6F12**	5′-	ACACTCTTTCCCTACACGACGCTCTTCCGATCTACTTCAACGCGARGAACCTTACC	-3′
**V6R1**	5′-	CTCGGCATTCCTGCTGAACCGCTCTTCCGATCTATAGCGACAACACGAGCTGACGAC	-3′
**V6R2**	5′-	CTCGGCATTCCTGCTGAACCGCTCTTCCGATCTAGGGTACAACACGAGCTGACGAC	-3′
**V6R3**	5′-	CTCGGCATTCCTGCTGAACCGCTCTTCCGATCTTTCATACAACACGAGCTGACGAC	-3′
**V6R4**	5′-	CTCGGCATTCCTGCTGAACCGCTCTTCCGATCTGATCGTACAACACGAGCTGACGAC	-3′
**V6R5**	5′-	CTCGGCATTCCTGCTGAACCGCTCTTCCGATCTGCCCGTACAACACGAGCTGACGAC	-3′
**V6R6**	5′-	CTCGGCATTCCTGCTGAACCGCTCTTCCGATCTCTGTCACAACACGAGCTGACGAC	-3′
**V6R7**	5′-	CTCGGCATTCCTGCTGAACCGCTCTTCCGATCTCACGTACAACACGAGCTGACGAC	-3′
**V6R8**	5′-	CTCGGCATTCCTGCTGAACCGCTCTTCCGATCTCGTACGACAACACGAGCTGACGAC	-3′
**V6R9**	5′-	CTCGGCATTCCTGCTGAACCGCTCTTCCGATCTGGACACAACACGAGCTGACGAC	-3′
**V6R10**	5′-	CTCGGCATTCCTGCTGAACCGCTCTTCCGATCTTAGAACAACACGAGCTGACGAC	-3′
**V6R11**	5′-	CTCGGCATTCCTGCTGAACCGCTCTTCCGATCTTCATACAACACGAGCTGACGAC	-3′
**V6R12**	5′-	CTCGGCATTCCTGCTGAACCGCTCTTCCGATCTACTTACAACACGAGCTGACGAC	-3′
**PCRF1**	5′-	AATGATACGGCGACCACCGAGATCTACACTCTTTCCCTACACGACGCTCTTCCGATCT	-3′
**PCRR1**	5′-	CAAGCAGAAGACGCCATACGAGATCGGTCTCGGCATTCCGTCTGAACCGCTCTTCCGATCT	-3′

Table S4. *Primers used to create V6 16S rRNA library for sequencing.* V6F1-12 and V6R1-12 were used to initial PCR. PCRF1/PCRR1 were used for second stage PCR.

Disease Models & Mechanisms • Supplementary information
